# The Potential of Electrospun Meshes in Postoperative Pain Management

**DOI:** 10.3390/pharmaceutics18050538

**Published:** 2026-04-28

**Authors:** Christodoulos Chrysanthou, Kenigen Manikion, Constantinos Voniatis

**Affiliations:** 1 Laboratory of Nanochemistry, Department of Biophysics and Radiation Biology, Semmelweis University, Tűzoltó Street 37-47, H-1094 Budapest, Hungary; 2 Department of Surgery, Transplantation and Gastroenterology, Semmelweis University, Üllői Street 78, H-1082 Budapest, Hungary

**Keywords:** surgical implant, enhanced recovery, surgical regeneration, nanotechnology

## Abstract

Postoperative pain management (POPM) remains a major clinical challenge despite its vital importance in reducing surgical stress, enabling early mobilization, and limiting postoperative complications. Conventional analgesic strategies are often constrained by short drug half-lives, repeated dosing requirements, systemic adverse effects, and the risk of opioid-related toxicity or dependence. These limitations suggest that the mode of drug delivery, in addition to drug selection itself, is a critical determinant of therapeutic performance. In this context, electrospun fibrous meshes represent a promising platform for localized and sustained analgesic delivery. Their high surface-area-to-volume ratio, tuneable porosity, broad polymer compatibility, and capacity to incorporate single or multiple bioactive agents make them attractive candidates for postoperative applications. This review summarizes recent advances in electrospun meshes for POPM, with particular emphasis on fabrication strategies, polymer selection, drug incorporation approaches, drug-release behaviour, biological performance, and translational challenges.

## 1. Introduction

**Figure 1:**
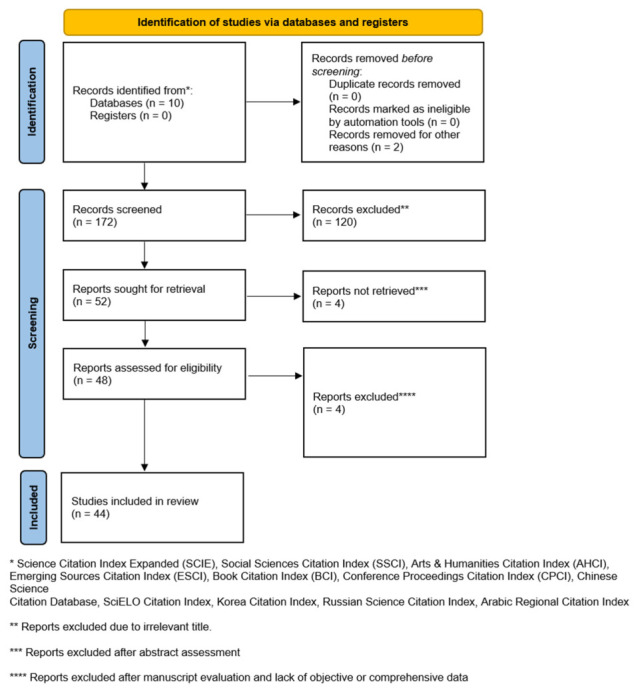
PRISMA 2020 flowchart of the current literature review.

In the current era of surgery, Enhanced Recovery After Surgery (ERAS) protocols have become a cornerstone of evidence-based perioperative care across surgical specialties. ERAS integrates preoperative optimization, intraoperative management, and postoperative recovery strategies to reduce surgical stress, accelerate rehabilitation, and shorten hospital stay [1]. Within this framework, multimodal analgesia is a key component of postoperative recovery [2,3]. By combining analgesic agents and techniques with complementary mechanisms of action, multimodal analgesia aims to improve pain control while reducing the adverse effects associated with high-dose single-agent therapy. Despite these advances, important limitations remain. Many currently used analgesics have short half-lives and therefore require repeated administration, whether by intermittent intravenous dosing, patient-controlled analgesia, or continuous epidural infusion [4]. Repeated dosing increases the risk of systemic adverse effects and catheter-related complications, including infection, dislodgement, and patient discomfort. In addition, prolonged administration may promote tolerance, dose escalation, and reduced clinical effectiveness. From a practical perspective, continuous monitoring and repeated dosing also increase the workload for clinical staff and may restrict patient mobility during the early recovery period. To address these limitations, increasing attention has been directed toward localized and sustained drug delivery strategies. Among these, electrospun meshes are particularly attractive because of their tuneable physicochemical properties, mechanical flexibility, and broad applicability in biomedical engineering [5]. Electrospun systems can also be designed to incorporate one or multiple therapeutic agents within a single fibrous platform [6]. In the following review, we briefly review the background on multimodal analgesia and present recent advances in electrospun membranes intended for pain management. We focus on physicochemical characterization, drug-release kinetics, and biological evaluation, without which clinical application is not feasible. Reporting of this review was performed according to the standards of the Preferred Reporting Items for Systematic Review and Meta-Analysis (PRISMA), as seen below (Figure 1). The structured systematic literature search and study selection process can be found in the Supplementary Materials.

## 2. Brief Overview of Postoperative Pain Management (POPM)

### 2.1. The Importance of Postoperative Pain

Effective postoperative pain control is critical for several reasons. Inadequately managed pain is associated with delayed recovery, increased complication rates, transition to chronic postsurgical pain, and psychological distress [7,8]. Firstly, severe postoperative pain can trigger a systemic stress response characterized by elevated heart rate, blood pressure, and cortisol levels, which may impair healing, exacerbate inflammation, and increase the risk of postoperative complications [9]. Secondly, inadequate pain control during the early postoperative period has been associated with an increased risk of chronic pain development [7,10,11].

Although the underlying mechanisms remain incompletely understood, accumulating evidence suggests that poorly controlled acute nociceptive input may promote central sensitization and persistent alterations in pain-processing pathways [11,12]. These changes may result in long-term hypersensitivity and chronic pain syndromes that persist for months or even years after surgery. In turn, persistent pain may adversely affect psychological well-being and contribute to anxiety, depression, and reduced quality of life [13]. This clinical problem further supports the development of drug delivery systems capable of providing sustained and localized analgesia during the early postoperative period. Furthermore, inadequate pain control may prolong hospitalization, increase exposure to nosocomial complications, and add further burden to both patients and healthcare systems [14], while exposing patients to further risks and hospitals to additional costs.

Finally, optimized pain control is crucial as it enables patients to enter early mobilization. In other words, patients can engage in fundamental recovery activities such as deep breathing, urination (without the use of a urinary catheter), eating and drinking, bowel movement, and physical therapy. The objective of early mobilization is to reduce complications such as thromboembolic events and hospital-acquired infections (e.g., deep vein thrombosis, surgical site infections, and pneumonia) while enhancing the healing process [15,16].

### 2.2. Multimodal Analgesia

**Figure 2:**
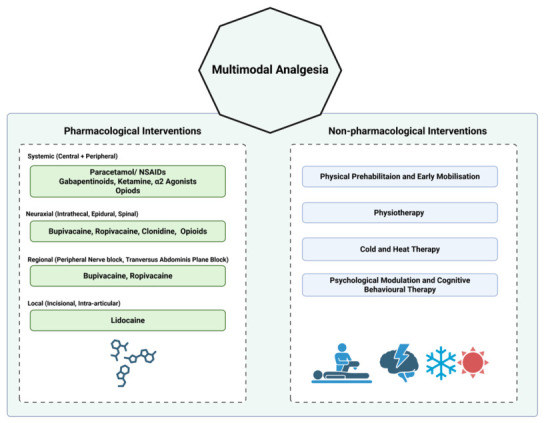
Schematic of multimodal analgesia. Created in BioRender. Voniatis, C. (2026) https://BioRender.com/uuukn25, accessed on 20 April 2026.

Multimodal analgesia is a pain-management strategy that combines drugs, regional techniques, and supportive non-pharmacological interventions to target different mechanisms of postoperative pain. Its primary objective is to improve analgesic efficacy while reducing the dose-related adverse effects of any single agent. Although multimodal analgesia is most commonly discussed in postoperative care, similar principles are also applied in chronic pain management [17]. It should be emphasised that multimodal analgesia does not only imply the use of multiple pharmaceutical agents. As seen in Figure 2, non-pharmacological methods, including physiotherapy, physical therapy, cold and heat therapy, and even cognitive behavioural therapy, are also incorporated [18,19,20]. By integrating complementary modalities, multimodal analgesia can reduce opioid requirements and improve functional recovery after surgery [21]. Non-pharmacological interventions are acknowledged here but are beyond the main scope of the present review.

### 2.3. Drug Administration

**Figure 3:**
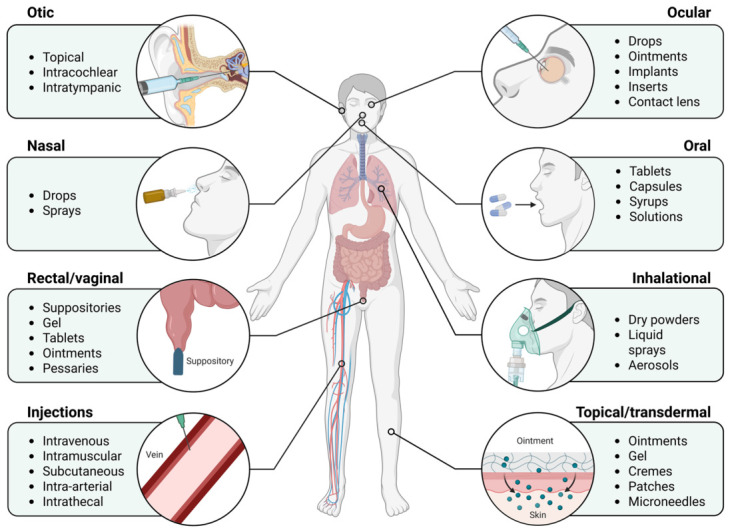
Options for drug administration (adapted from Tucak-Smajić, A.). Created in BioRender. Voniatis, C. (2026) https://BioRender.com/u2mfpui, accessed on 20 April 2026.

The route of drug administration is a key consideration in postoperative pain management and is typically selected according to the type of surgical procedure, the expected pain intensity, and patient-specific clinical factors. In clinical practice, analgesic delivery strategies vary across surgical specialties and perioperative contexts. For example, intravenous administration is commonly used for immediate postoperative analgesia and for patients who cannot tolerate oral intake, such as those undergoing major abdominal or gastrointestinal surgery. Epidural or spinal analgesia is frequently employed in thoracic, abdominal, and major orthopaedic procedures where regional blockade can provide effective segmental pain control. Local infiltration and peripheral nerve blocks are widely applied in orthopaedic, trauma, and ambulatory surgeries to achieve targeted analgesia while minimizing systemic drug exposure. Once oral intake is restored, oral analgesics are typically introduced for ongoing pain management during recovery. Other delivery routes, including inhalational, transdermal, and rectal administration, may be used in specific clinical settings but remain less common in routine postoperative analgesia. It is worth mentioning that not all medications are available in every possible formulation. Consequently, the administration route must be selected based on the available pharmaceutics, the patient’s condition, and their medical history. A summary of the main administration routes used in postoperative pain management (Figure 3) and their clinical considerations is provided in Table 1.

### 2.4. Pharmaceutical Agents

#### 2.4.1. Opioids

Drugs such as morphine, fentanyl, and oxycodone are the most potent analgesics used in POPM. They act primarily through the μ-opioid receptors in the central nervous system, inhibiting pain transmission [33]. Opioids are particularly effective in managing moderate to severe pain but are associated with significant side effects, including respiratory depression, constipation, and the potential for addiction or misuse [34]. Despite these risks, opioids remain a cornerstone for managing severe acute postoperative pain.

#### 2.4.2. Nonsteroidal Anti-Inflammatory Drugs (NSAIDs)

NSAIDs, including ibuprofen, naproxen, and diclofenac, are commonly used in the management of mild to moderate postoperative pain. These drugs exert their analgesic effects by inhibiting cyclooxygenase (COX) enzymes, which are responsible for the synthesis of prostaglandins, compounds that mediate inflammation and pain [35]. NSAIDs are effective in reducing both pain and inflammation, making them useful in conditions where inflammation is a contributing factor. However, long-term use or high doses can lead to adverse effects such as gastrointestinal bleeding, renal and cardiovascular toxicity [36,37].

#### 2.4.3. Acetaminophen

Acetaminophen (paracetamol) is widely used for mild to moderate postoperative pain, often in combination with opioids or NSAIDs. Its exact mechanism involves inhibition of COX enzymes in the brain, as well as modulation of the endocannabinoid system [38]. Acetaminophen is generally well-tolerated and has a lower risk of gastrointestinal and renal side effects compared to NSAIDs. However, overdose can lead to severe liver toxicity, making appropriate dosing essential [39].

#### 2.4.4. Local Anaesthetics

Local anaesthetics, such as lidocaine, bupivacaine, and ropivacaine, are used in POPM, particularly in the context of regional anaesthesia. They block sodium channels in nerve fibres, preventing the propagation of pain signals from the site of surgery to the CNS [40]. Local anaesthetics can be administered via epidural or peripheral nerve blocks, providing effective analgesia without the need for systemic drugs. These agents are particularly useful for pain following procedures such as orthopaedic surgery, caesarean sections, and abdominal surgery [41].

#### 2.4.5. Adjuvant Drugs

Adjuvants, such as anticonvulsants (e.g., gabapentin and pregabalin) and antidepressants (e.g., amitriptyline and duloxetine), are increasingly recognized for their role in managing postoperative pain, particularly neuropathic pain. Anticonvulsants work by stabilizing neuronal membranes and inhibiting excitatory neurotransmitter release, while antidepressants can modulate pain through the serotonin and norepinephrine systems [42]. These drugs are used in combination with other analgesics to provide more comprehensive pain relief and are particularly beneficial in patients experiencing chronic or neuropathic pain after surgery [43].

A brief overview of commonly used drugs relevant to this review and their features can be found in Table 2.

### 2.5. Clinical Implications of Current Analgesic Strategies

Current evidence strongly supports multimodal analgesia as the preferred strategy for postoperative pain management, combining agents with complementary mechanisms of action to improve analgesic efficacy while minimizing adverse effects. This approach typically integrates non-opioid analgesics such as NSAIDs and acetaminophen, opioids for moderate-to-severe pain, and adjuvant agents including gabapentinoids or antidepressants, alongside regional anaesthesia techniques. Such combinations have been shown to reduce opioid consumption, improve pain control, and enhance postoperative recovery [52,53,54]. In clinical practice, analgesic selection should be procedure-specific and patient-centred. Intravenous opioids and non-opioid analgesics are commonly used in the immediate postoperative period, particularly in patients undergoing major abdominal or gastrointestinal surgery where oral intake is not feasible. Regional techniques, including epidural and spinal anaesthesia with local anaesthetics (e.g., bupivacaine, ropivacaine), are widely applied in thoracic, abdominal, and major orthopaedic procedures to provide effective segmental pain control. Peripheral nerve blocks and local anaesthetic infiltration (e.g., lidocaine, bupivacaine) are frequently used in orthopaedic and ambulatory surgery to achieve targeted analgesia while minimizing systemic exposure. Once oral intake is restored, oral NSAIDs, acetaminophen, and opioid combinations are typically introduced for continued pain management during recovery [52,53,54].

Despite these established pharmacological strategies, current approaches remain limited by short drug half-lives, systemic distribution, and the need for repeated administration, which can increase the risk of adverse effects such as gastrointestinal toxicity, sedation, or respiratory depression. These limitations highlight the need for localized and sustained drug delivery systems capable of maintaining therapeutic concentrations at the surgical site while minimizing systemic exposure.

Several advanced drug-delivery strategies have been explored to improve postoperative pain management, including polymeric films, hydrogels, liposomal formulations, and nanoparticle-based systems. Among these, electrospinning has emerged as a particularly promising fabrication method because it enables the production of highly porous fibrous structures with large surface-area-to-volume ratios, a tuneable architecture, and controllable drug incorporation. These features make electrospun meshes attractive candidates for localized and sustained postoperative analgesic delivery.

## 3. A Brief Overview of Electrospun Meshes

### 3.1. Electrospinning

Electrospinning is a widely used technique for fabricating polymeric meshes composed of micro- or nanoscale fibres. Owing to its versatility, it has been extensively applied in pharmaceutical science, drug delivery, wound healing, and tissue engineering [55,56]. The method is compatible with a broad range of polymers, including synthetic and natural materials as well as water-soluble and water-insoluble systems, making it a flexible platform for therapeutic incorporation and controlled release [57,58].

**Figure 4:**
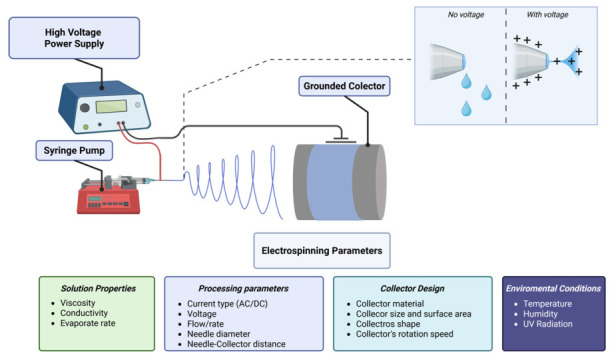
Schematic of the electrospinning technique. Created in BioRender. Voniatis, C. (2026) https://BioRender.com/6btkadl, accessed on 20 April 2026.

Briefly, a polymer solution is loaded into a syringe fitted with a metallic needle connected to a high-voltage power supply, typically in the range of 10–30 kV. When the electrostatic force overcomes the surface tension of the solution, a Taylor cone forms at the needle tip and ejects a charged jet toward a grounded collector [55]. During flight, the solvent evaporates and solid fibres are deposited onto the collector, generating either randomly oriented or aligned fibrous mats depending on collector design [57] (Figure 4).

The morphology and properties of the resulting fibres are influenced by several parameters, including polymer concentration, applied voltage, flow rate, needle-to-collector distance, and environmental conditions. Proper control of these parameters is critical to achieve uniform fibres with predictable structural and functional characteristics [56,59].

### 3.2. Electrospinning Parameters

The fabrication of electrospun meshes is governed by a complex interplay of physicochemical and processing parameters that collectively determine fibre morphology and size, drug distribution, and release kinetics [55,56,57]. It is paramount to mention that these parameters are not independent; rather, they are dynamic and interdependent. In other words, modification of one often necessitates compensatory adjustment of others (Figure 4). A detailed understanding of these interrelationships is essential for producing reproducible fibres with controlled therapeutic performance [56,57].

#### 3.2.1. Solution Properties

The polymer concentration serves as a primary determinant of both fibre diameter and drug encapsulation efficiency. At higher concentrations, enhanced polymer chain entanglement improves jet stability, resulting in thicker, smoother fibres capable of entrapping higher drug loads. Conversely, low concentrations often yield thinner fibres but can induce jet instability, bead formation, and heterogeneous drug distribution. Viscosity, which is a function of polymer type, molecular weight, and solvent composition, critically influences jet continuity and fibre uniformity. Solutions that are too dilute tend to generate electrosprayed droplets instead of fibres, producing surface-localized drug accumulation and rapid burst release. In contrast, highly viscous solutions can obstruct flow and yield irregular fibre morphologies, compromising both encapsulation uniformity and mechanical properties. Furthermore, the solvent system plays a dual role by dissolving both the polymer and ensuring adequate volatility for solvent evaporation during jet flight [55,59,60].

In addition, solution ionic strength and pH can also influence fibre morphology and drug release behaviour. Changes in ionic strength alter the electrical conductivity of the spinning solution, which can affect jet stability and fibre diameter. Higher ionic strength typically increases the charge density in the jet, leading to greater elongation and potentially thinner fibres. Similarly, solution pH can modify polymer ionization, intermolecular interactions, and drug solubility within the spinning solution. These factors may ultimately influence the drug distribution within the fibres and subsequent release kinetics.

More importantly, solvents must also be compatible with the pharmaceutical agent. Solvent polarity and evaporation rate directly affect drug–polymer miscibility, thereby dictating whether the incorporated drug is molecularly dispersed, amorphous, or phase-separated within the fibre matrix. Inappropriate solvent selection can lead to drug crystallization or uneven loading, resulting in erratic release profiles and poor bioavailability [60].

#### 3.2.2. Processing Parameters

Among the key electrospinning variables, applied voltage governs jet initiation and stretching dynamics. An optimal voltage range must balance the electrostatic and viscoelastic forces to sustain a stable jet. Insufficient voltage fails to form a continuous jet, whereas excessive voltage promotes jet fragmentation, leading to bead formation and non-uniform drug encapsulation. The needle inner diameter influences jet stability and fibre morphology by controlling the initial volume and velocity of the polymer solution exiting the spinneret. Smaller needle diameters tend to enhance electrostatic stretching, yielding thinner fibres; however, excessively narrow gauges can induce clogging, pressure build-up, and erratic jet formation, particularly when high-viscosity polymer solutions are used [61].

The solution flow rate regulates the rate of polymer delivery to the Taylor cone. Low flow rates typically favour the formation of uniform fibres with consistent drug dispersion due to sufficient solvent evaporation and elongation time. In contrast, high flow rates increase droplet size, reduce flight time, and often lead to bead formation, fibre fusion, and incomplete solvent removal. Therefore, the optimal flow rate must be carefully matched with voltage, solution viscosity, and needle gauge to ensure continuous, stable jet formation and uniform fibre morphology [61,62].

The needle-to-collector distance determines the duration of jet travel and, consequently, the time available for polymer elongation and solvent evaporation. A longer distance generally promotes finer, more uniform fibres and complete solvent removal—factors that stabilize drug distribution within the matrix. Conversely, short working distances can result in incomplete solvent evaporation and partial drug migration toward the fibre surface, contributing to an undesired initial burst effect [60].

#### 3.2.3. Collector Design

The collector configuration dictates fibre alignment, packing density, and surface morphology—all of which impact drug release behaviour. Static collectors yield randomly oriented mats with isotropic porosity, typically promoting faster and more homogeneous diffusion. In contrast, rotating drum or mandrel collectors generate aligned fibres, introducing anisotropy that can modulate diffusion pathways and mechanical strength. Adjusting collector geometry and rotational speed provides a powerful means of tailoring drug release kinetics and scaffold performance for specific therapeutic contexts, such as wound healing, localized chemotherapy, or tissue regeneration [63].

#### 3.2.4. Environmental Conditions

Ambient humidity and temperature strongly influence solvent evaporation rates and fibre solidification kinetics, especially in drug-loaded systems. Elevated humidity levels can induce pore formation or phase separation within fibres, accelerating diffusion-mediated drug release. Conversely, low humidity may cause rapid solvent loss and premature jet solidification, entrapping the drug heterogeneously. Controlled airflow around the electrospinning zone can further stabilize the jet and enhance uniform fibre deposition. In some cases, UV radiation is used for in situ polymer crosslinking or sterilisation purposes [64].

### 3.3. Drug Incorporation in Electrospun Nanofibres

As noted above, a key advantage of electrospinning as a fabrication technique lies in its remarkable capacity to incorporate bioactive compounds directly into nanofibrous matrices [57]. This feature enables the creation of multifunctional meshes that can serve as advanced drug delivery systems. Incorporation of drugs within electrospun fibres can prolong drug release and improve release stability. Furthermore, the system can integrate multiple polymers, enable multi-drug composite formulations, and precisely control staged release profiles [5].

#### 3.3.1. Pre-Spinning Inclusion

**Figure 5:**
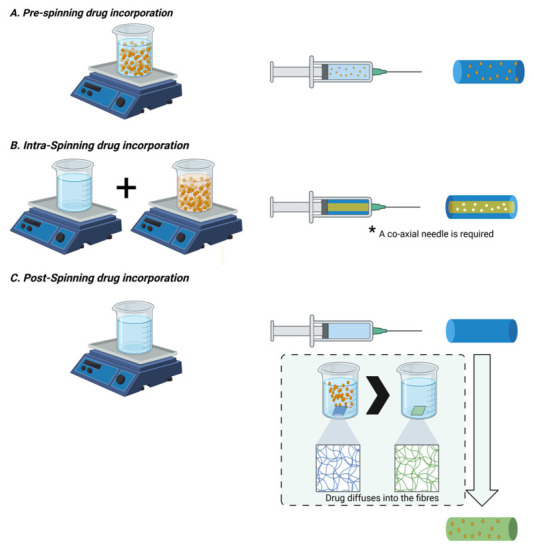
Drug incorporation strategies in electrospinning. Created in BioRender. Voniatis, C. (2026) https://BioRender.com/jyqxqld, accessed on 20 April 2026.

This approach involves dissolving or dispersing the drug directly into the polymer solution before electrospinning (Figure 5A) [65]. It is the most straightforward and widely used method because of its simplicity and the relatively uniform drug distribution achieved throughout the fibre matrix. The drug is encapsulated as the polymer jet solidifies during the electrospinning process, resulting in molecular-level dispersion or amorphous entrapment within the fibres [66].

However, several factors must be considered to ensure efficient encapsulation and stability. The drug–polymer–solvent compatibility determines whether the drug remains molecularly dispersed or undergoes phase separation during fibre formation [60]. Poor solubility or interactions between the components can lead to drug crystallization, heterogeneous loading, or surface enrichment, altering drug release kinetics and reproducibility [67]. Furthermore, pre-spinning inclusion is particularly suitable for hydrophobic drugs soluble in organic solvents and compatible with hydrophobic polymers such as polycaprolactone (PCL) or poly(lactic acid) (PLA) [68].

#### 3.3.2. Intra-Spinning Incorporation

Intra-spinning strategies (Figure 5B), such as coaxial and emulsion electrospinning, provide more sophisticated control over drug localization within the fibres [55]. In coaxial electrospinning, two concentric solutions are simultaneously electrospun—typically a drug-containing core and a polymeric shell. This configuration produces core–shell fibres that protect sensitive drugs (e.g., proteins or growth factors) from environmental exposure and provide sustained or delayed release through diffusion barriers in the outer layer [69].

Similarly, emulsion electrospinning disperses the drug phase as droplets within an immiscible polymer solution, enabling controlled encapsulation of hydrophilic drugs within hydrophobic matrices [70]. These methods are especially valuable for drugs requiring protection from harsh solvents or moisture and for systems demanding multi-stage or biphasic release kinetics [56]. The structure–property relationship in these fibres is defined by shell thickness, core viscosity, and interfacial stability. Due to the additional equipment required (e.g., a coaxial needle) and the associated challenges in reproducibility, these techniques have not yet reached scaled-up industrial settings [58].

#### 3.3.3. Post-Spinning Functionalization

Post-spinning drug incorporation involves introducing the therapeutic agent after fibre fabrication, typically by surface adsorption, solvent swelling, physical soaking, or covalent immobilization (Figure 5C). This approach is particularly advantageous when the drug is heat- or solvent-sensitive since it avoids direct exposure during electrospinning [71].

The simplest post-spinning method is soaking or immersion, where fibres are exposed to a drug solution under controlled conditions, allowing the drug to diffuse into the polymer matrix or adsorb onto the surface. Although this method offers operational flexibility, drug loading is often limited, and surface-bound molecules may cause burst release upon initial contact with physiological fluids [72]. More sophisticated methods, such as plasma treatment, layer-by-layer assembly, or chemical conjugation, can be used to functionalize the fibre surface with bioactive agents, achieving prolonged and tuneable release profiles while preserving biological activity [73].

#### 3.3.4. Influence on Drug Release Behaviour

The drug release behaviour of electrospun nanofibres arises from the intricate interplay among material composition, fibre morphology, and processing conditions. Critical determinants include polymer chemistry, drug solubility, fibre diameter, porosity, and the spatial distribution of the active compound within the fibrous matrix [74]. When the drug is molecularly dispersed within the polymer prior to electrospinning, release typically follows a diffusion-controlled mechanism governed by molecular transport through the polymer network. In contrast, surface-enriched or post-coated systems exhibit a characteristic initial burst release, reflecting rapid desorption or dissolution of drug species located near or on the fibre surface, followed by a slower diffusion or degradation-mediated phase [72].

The physicochemical interaction between the drug and the polymer matrix during electrospinning is crucial. Drug–polymer compatibility can affect drug dispersion within the fibres, crystallization behaviour, and long-term stability of the formulation. Favourable interactions between the polymer and drug molecules may promote amorphous drug dispersion and improved encapsulation efficiency, whereas poor compatibility can lead to phase separation or surface localization of the drug. Such effects may significantly influence the initial burst release and the long-term diffusion profile of the therapeutic agent.

Additionally, non-covalent interactions play a key role in stabilizing drug-loaded electrospun fibres and modulating drug release behaviour. Interactions such as hydrogen bonding, π–π stacking, and hydrophobic interactions can influence the spatial distribution of drug molecules within the polymer matrix. For example, hydrogen bonding between drug functional groups and polymer chains may stabilize the drug within the fibre structure, reducing burst release and enabling more sustained diffusion. Similarly, hydrophobic interactions between drug molecules and hydrophobic polymer domains can promote stronger drug retention within the matrix. Understanding these intermolecular interactions is therefore critical for designing electrospun systems with predictable and controllable drug release profiles.

Advanced core–shell architectures, fabricated via coaxial electrospinning, enable finely tuned multiphase release kinetics. In these systems, the outer polymer sheath modulates permeability, hydration, and degradation rate, thereby providing precise temporal control over lag time and sustained release [75]. This design flexibility facilitates sequential or prolonged drug delivery profiles suited to complex therapeutic regimens.

The polymer degradation rate constitutes a secondary regulatory mechanism. Biodegradable polymers such as PCL, PLA, and poly(lactic-co-glycolic acid) (PLGA) exhibit degradation-coupled release behaviour, producing biphasic or triphasic profiles ideal for long-term applications [76].

Ultimately, the interdependence of formulation, processing, and environmental parameters dictates the dominant release mechanism. Mastery of these relationships permits the rational design of electrospun drug delivery systems with predictable and tuneable kinetics [74]. Continued advances in polymer–drug compatibility, solvent engineering, and real-time process monitoring are expected to enhance the reproducibility, scalability, and clinical translation of electrospun therapeutic meshes for both localized and systemic drug delivery.

## 4. Advances in Electrospun Membranes for Postoperative Pain Management

### 4.1. Mesh Fabrication Strategies

Advances in electrospun membranes for POPM can be broadly categorized into two strategies. The first focuses on optimizing single-component electrospun meshes through control of polymer composition and fibre morphology to achieve predictable and sustained release of analgesic and anti-inflammatory agents. The second strategy involves multicomponent electrospun systems designed for coordinated or sequential delivery of multiple therapeutics. Such functionality is commonly enabled by single-needle, coaxial, and multilayer electrospinning architectures, which permit spatial separation of drugs within distinct fibre compartments. Coaxial electrospinning, in particular, can protect labile compounds within the fibre core while the surrounding shell enables diffusion-controlled release (and, depending on polymer chemistry, erosion-modulated sustained delivery). Collectively, these approaches aim to generate biodegradable and biocompatible membranes capable of localized, long-term pain control while supporting tissue regeneration and minimizing systemic exposure and associated adverse effects.

### 4.2. Pharmaceuticals

Local anaesthetics represent the most extensively investigated drug class, with lidocaine, bupivacaine, ropivacaine, benzocaine, and tramadol repeatedly reported across formulations [77,78,79,80,81,82,83,84,85,86,87,88,89,90,91,92,93,94,95,96,97,98,99,100]. Lidocaine, due to its rapid onset and moderate duration of action, is incorporated into both hydrophilic and hydrophobic polymer matrices, either alone or combined with antibiotics, anti-inflammatory agents, or growth factors [78,83,89,90,91,92,93,94]. In contrast, longer-acting anaesthetics such as bupivacaine and ropivacaine are more frequently embedded within biodegradable polyesters—most notably PLGA and PCL—to support sustained local delivery over days to weeks [77,84,85,86,87,88]. These systems are typically evaluated in rodent or rabbit models, where prolonged local delivery is reported to reduce systemic exposure and extend the duration of pharmacological effect [77,78,83,84,85,86,87,88].

Non-steroidal anti-inflammatory drugs (NSAIDs)—including diclofenac, ibuprofen, ketorolac, indomethacin, aceclofenac, meloxicam, and celecoxib—constitute the second major drug category [79,87,88,96,97,98,99,100,101,102,103,104,105,106,107,108,109,110,111,112,113]. NSAIDs are frequently paired with local anaesthetics to support multimodal pain control, combining early neural blockade with sustained suppression of inflammatory mediators [87,88,91]. When used alone, NSAIDs are often incorporated into hydrophilic polymer systems to facilitate rapid diffusion and early therapeutic availability; however, polyester-based matrices are increasingly used to extend release duration and mitigate burst effects [80,96,97].

Adjuvant and multifunctional agents—including antibiotics (vancomycin, ceftazidime, mupirocin, gentamicin), growth factors (human EGF), hormones (estradiol), and bioactive small molecules (curcumin, capsaicin, borneol, papaverine, lovastatin)—reflect a growing emphasis on integrating infection control, tissue regeneration, and vascular modulation alongside analgesia [81,89,90,94,112]. These agents are rarely delivered as monotherapies and are more commonly incorporated into multi-drug or layered systems intended to provide sequential or complementary effects during postoperative healing.

### 4.3. Polymer Selection and Drug Release Modulation

Following drug selection, polymer choice is used to tune release kinetics and mechanical performance. Hydrophilic polymers such as Poly(vinylpyrrolidone) (PVP), poly(vinyl alcohol) (PVA), polyethylene oxide (PEO), carboxymethylcellulose (CMC), and alginate are frequently employed for drugs requiring rapid onset, including lidocaine, NSAIDs, and acetaminophen [79,82,93,101,102,103,104,110,114]. Release from these matrices is dominated by swelling and dissolution, enabling rapid diffusion-driven delivery suited to acute pain control but inherently prone to burst effects.

In contrast, biodegradable polyesters—particularly PLGA and PCL—are preferentially selected for sustained delivery of potent anaesthetics and combination therapies [77,80,83,84,85,86,87,88,89,90,91,94,96,97]. PLGA-based systems commonly exhibit multi-phase kinetics, with polymer composition and molecular weight enabling modulation of release duration from days to approximately one month. PCL-based membranes generally degrade more slowly and provide higher mechanical stability, supporting prolonged delivery and improved handling in surgically relevant environments.

Hybrid and blended polymer systems—combining hydrophilic and hydrophobic components or incorporating natural polymers such as gelatin, chitosan, silk fibroin, or collagen—are increasingly used to reconcile competing requirements for rapid onset, sustained delivery, and biological integration [81,86,92,109,112]. These blends can enable biphasic or staged release while improving wettability, cell interaction, and tissue compatibility.

More advanced formulations employ multilayered or core–shell electrospun architectures to spatially segregate drugs and polymers with distinct release characteristics [78,86,89,90,91,94]. This approach supports early release of surface-associated analgesics followed by sustained delivery of anti-inflammatory, antimicrobial, or regenerative agents from inner compartments, aligning therapeutic availability with the temporal progression of postoperative pain and wound healing.

Post-fabrication, electrospun membranes are typically characterized using scanning electron microscopy (SEM) to assess fibre uniformity and diameter distribution, Fourier-transform infrared spectroscopy (FTIR) to confirm chemical integrity and drug–polymer compatibility, and uniaxial tensile testing to quantify flexibility and strength under physiological loading. Hydrophilicity and degradation behaviour are commonly evaluated by contact-angle measurements and mass-loss assays, while in vitro release testing—most often conducted in phosphate-buffered saline (PBS, pH 7.4)—is used to define release kinetics (typically quantified by UV–Vis or HPLC-based methods). As indicated in Table 3, however, most studies prioritize release profiling and provide limited, non-site-specific mechanical testing and/or biological evaluation. This imbalance hampers cross-study comparability and constrains translation from laboratory development to clinically validated devices and routine clinical use.

Despite the diversity of drug–polymer combinations explored, most studies emphasise release kinetics over comprehensive mechanical and biological validation. Mechanical testing is inconsistently reported, and biological evaluation is often limited to short-term pharmacokinetic measurements in small animal models. Consequently, while drug-driven polymer design has enabled increasingly sophisticated POPM systems, the lack of standardised performance benchmarks and clinically relevant endpoints continues to constrain translational advancement.

### 4.4. Specific Drug Release

Table 4 depicts a wide spectrum of drug-release profiles from electrospun membranes developed for POPM, ranging from rapid dissolution within minutes to sustained delivery extending beyond one month. This variability arises primarily from differences in polymer hydrophilicity, degradation kinetics, matrix architecture (single-layer, multilayer, or composite), and the physicochemical properties of the encapsulated drug, including solubility, molecular weight, and ionization behaviour.

Among fast-releasing systems, hydrophilic polymers such as PVP, PVA, and PEO dominate due to their high water solubility and rapid hydration in aqueous environments [79,82,93,100,101,103,104,114]. Ibuprofen-loaded PVP fibres, for example, demonstrated complete drug release within 30–1000 min depending on fibre composition, enabling classification into fast- and sustained-release variants [79]. Similarly, acetaminophen embedded in PEO/alginate nanofibres achieved near-total diffusion within approximately 3 h, driven by polymer swelling and high water uptake [114].

When combined in multilayer constructs (PEO/PVA/alginate), dual-drug systems such as acetaminophen/gabapentin exhibited two distinct release phases: rapid initial diffusion from the PEO layer followed by slower release from the crosslinked PVA–alginate matrix, extending delivery up to approximately 18 h [114]. These fast-dissolving platforms are therefore well suited for immediate postoperative pain relief, where rapid analgesic onset is clinically desirable.

In contrast, hydrophobic and biodegradable polyesters such as PLGA, PCL, and PLLA enable extended drug release spanning weeks through combined diffusion- and erosion-controlled mechanisms [77,80,83,84,85,86,87,88,89,91,94,96,97]. Ibuprofen embedded in PLGA nanofibres showed gradual diffusion with complete release after approximately 60 days, reflecting slow matrix erosion and limited water penetration [80]. Bupivacaine and lidocaine incorporated into PLGA membranes followed characteristic triphasic kinetics, consisting of a short burst phase (<24 h), a diffusion-dominated plateau lasting 1–2 weeks, and a degradation-controlled terminal phase extending up to 30–35 days [83,84,85].

Similar release behaviour was observed in PLGA/PEG composites co-loaded with bupivacaine and acetaminophen, where incorporation of PEG increased matrix hydrophilicity and shortened the diffusion-dominated phase, reducing total release duration to approximately 14–20 days [85].

PCL-based systems demonstrated even more gradual kinetics due to their slower degradation rate. Curcumin-loaded PCL fibres released only ~25% of the payload within the first day and continued drug elution over more than 36 days [81]. Epinephrine- and lidocaine-loaded PCL meshes exhibited ratio-dependent release profiles extending up to 30 days [78]. Multilayer PCL/PLGA composites co-loaded with metronidazole, oestradiol, and lidocaine further exemplify staggered multi-drug delivery, with rapid antimicrobial release within the first 2 days, sustained anaesthetic elution for approximately 20 days, and extended hormonal delivery beyond 30 days [90].

Natural and hybrid polymer systems generally exhibited intermediate kinetics that combine rapid initial diffusion with moderate sustained phases [81,103,104,109,111,112,113]. Diclofenac and lidocaine incorporated into CMC/PEO or CMC/alginate/PEO scaffolds displayed biphasic release profiles, with approximately 25–30% of the dose released within the first hour followed by sustained diffusion extending beyond 50 h [103,104]. Meloxicam delivered from chitosan/PVA/hydroxyapatite films showed controlled release over approximately 25 h [111], whereas incorporation of chitosan into PCL matrices extended delivery beyond 14 days for meloxicam and mitomycin-C [112]. Similarly, indomethacin encapsulated in gelatin/PLCL nanofibres achieved progressive diffusion over 14 days, illustrating how blending hydrophilic biopolymers with degradable polyesters can moderate burst release while maintaining prolonged delivery [83].

The incorporation of cyclodextrins and surface modifiers markedly accelerated release from otherwise hydrophobic matrices. Naproxen complexed with β-cyclodextrin or hydroxypropyl-β-cyclodextrin in thermoplastic polyurethane (TPU) fibres achieved >90% release within 4 h, compared with release times exceeding 80 h for ethyl cellulose/PVP systems lacking cyclodextrins [101,102]. This acceleration is attributed to enhanced drug solubility and reduced crystallinity arising from host–guest complexation.

Environment-responsive behaviour was also observed in cellulose acetate systems loaded with benzocaine, where release was strongly pH-dependent—accelerating under alkaline conditions and slowing markedly under acidic environments [98,99]. In addition, PLLA/cellulose acetate butyrate blends loaded with borneol demonstrated composition-dependent release, with increasing CAB content extending release duration from under 2 h to approximately 8 h [83].

Systems designed for multidrug or multifunctional delivery frequently employed layered or composite architectures to achieve sequential release profiles [78,86,88,89,90,91,94,96,97]. PLGA/collagen scaffolds co-loaded with bupivacaine and doxycycline produced dual-peak kinetics, enabling early analgesic delivery followed by prolonged antimicrobial elution [86]. Similarly, PLGA membranes incorporating ketorolac, ceftazidime, and vancomycin exhibited rapid analgesic release within the first few days, followed by sustained antibiotic delivery extending beyond one month [96,97]. Comparable staged profiles were reported for celecoxib and bupivacaine in PLGA composites, with celecoxib released within days and bupivacaine persisting for up to four weeks [88].

Overall, these studies demonstrate a clear structure–function relationship between polymer composition and drug-release behaviour [77,78,79,80,81,82,83,84,85,86,87,88,89,90,91,92,93,94,95,96,97,98,99,100,101,102,103,104,105,106,107,108,109,110,111,112,113,114]. Hydrophilic matrices (PVP, PVA, PEO, and alginate) favour rapid diffusion-driven release, which is suited for early postoperative analgesia, whereas biodegradable polyesters (PLGA, PCL, and PLLA) support long-term, erosion-controlled delivery over weeks. Hybrid and multilayer constructs bridge these extremes, enabling staged or sequential release profiles that better align with the evolving pharmacological demands of POPM and tissue healing.

### 4.5. Limitations, Efficacy, Safety Considerations and Clinical Translation

#### 4.5.1. Limitations

Unequivocally, research on drug-releasing electrospun fibres is scientifically compelling. However, current evidence remains limited and heterogeneous, with most studies focusing on in vitro release profiling and short-term small-animal models. Standardised evaluation protocols are lacking, and clinically relevant endpoints such as drug release profiles, long-term safety, functional recovery, and sustained analgesic efficacy are inconsistently reported. In addition, variability in polymer systems, fabrication parameters, and experimental conditions hinders direct comparison across studies and limits reproducibility. These limitations must be considered when interpreting reported outcomes and highlight the need for more rigorous and standardised translational research.

#### 4.5.2. Efficacy Assessment

Compared with conventional dosage forms, electrospun meshes could provide several pharmacokinetic advantages related primarily to sustained local drug availability. As summarized in Table 2, commonly used analgesics such as morphine and ibuprofen exhibit plasma half-lives of approximately 2–4 h, while acetaminophen typically has a half-life of 2–3 h. Consequently, therapeutic pain control generally requires repeated dosing every 4–6 h, which increases cumulative systemic exposure and the risk of adverse effects. In contrast, electrospun meshes can maintain therapeutic drug release for substantially longer periods. As shown in Table 4, hydrophilic electrospun systems designed for rapid analgesic onset can release drugs within minutes to hours, comparable to immediate-release oral formulations. For example, acetaminophen incorporated into polyethylene oxide/sodium alginate nanofibres achieved 50% release within approximately 35 min and complete release within about 3 h [114]. Similarly, ibuprofen-loaded poly(vinyl pyrrolidone) fibres demonstrated >75% release within 5 min and complete release within 30 min, mimicking rapid-onset pharmaceutical formulations [79]. More importantly, meshes fabricated from biodegradable polyesters provide prolonged drug delivery far exceeding the duration achievable with conventional administration routes. PLGA nanofibres containing ibuprofen released 50% of the drug after approximately 24 days and more than 75% after 50 days, with complete release extending beyond 60 days [80]. Similarly, bupivacaine incorporated into PLGA electrospun membranes demonstrated sustained release for up to 35 days [84], while lidocaine-loaded PLGA fibres maintained measurable release for approximately 15 days [83]. Polycaprolactone-based systems can further extend release durations; for instance, curcumin-loaded PCL nanofibres exhibited only ~25% drug release within 24 h, continuing release for more than 36 days [81]. In vivo studies provide additional evidence of prolonged pharmacological activity. Ropivacaine-loaded PCL nanofibres implanted in Sprague–Dawley rats maintained detectable drug concentrations for up to 72 h post-surgery [77], whereas PLGA-based systems delivering bupivacaine or lidocaine maintained measurable plasma or tissue concentrations for 1–35 days depending on the formulation [83,84,85,86]. In comparison, conventional single-dose local anaesthetic injections generally provide analgesia for 1–12 h, and continuous infusion techniques such as epidural analgesia require catheter placement and continuous drug administration.

Nevertheless, several limitations remain when comparing electrospun meshes with conventional dosage forms.

#### 4.5.3. Safety Evaluation

Safety evaluation of these meshes should focus on biocompatibility, inflammatory responses, and systemic drug exposure. Electrospun scaffolds composed of biodegradable polymers such as PLGA, PCL, gelatin, chitosan, or alginate generally demonstrate favourable tissue compatibility and gradual degradation with minimal inflammatory responses in animal models [81,86,94]. Another safety advantage arises from reduced systemic exposure. Conventional systemic analgesics (i.e., NSAIDs and opioids) are associated with adverse effects including gastrointestinal toxicity, renal impairment, or respiratory depression due to high circulating drug concentrations [40,41,42,43,44]. By delivering drugs locally at the surgical site, electrospun meshes may reduce these systemic risks while maintaining effective analgesia. However, potential safety concerns include initial burst release, particularly in hydrophilic matrices where a considerable proportion of drug may be released within the first hours. For example, diclofenac-loaded CMC/PEO fibres released approximately 25–30% of the drug within the first hour, followed by sustained diffusion for more than 50 h [103,104]. While such burst effects may provide rapid analgesic onset, excessive initial release could transiently increase systemic exposure if not carefully controlled through polymer selection and fibre design.

#### 4.5.4. Translational Considerations

Despite positive preclinical findings, the translational evidence for electrospun drug-eluting meshes remains limited. Most available studies rely on small animal models, primarily rodents, with relatively short observation periods ranging from hours to a few weeks, as reflected in the studies summarized in Table 3. While these experiments provide important proof-of-concept data regarding drug release and short-term pharmacological activity, they do not adequately address the long-term safety, degradation behaviour, and tissue response expected in clinical applications. A notable gap in the current literature is the lack of large-animal studies and extended follow-up periods. Models such as rabbits, pigs, or sheep more closely replicate human surgical environments, implant dimensions, and tissue healing dynamics. Without such investigations, critical aspects—including long-term polymer degradation, chronic inflammatory response, implant stability, and sustained pharmacological efficacy—remain insufficiently evaluated.

#### 4.5.5. Future Prospects

From a technological perspective, continued refinement of advanced electrospinning architectures, including multilayer and core–shell fibres, may enable more precise temporal control of drug delivery and support staged therapeutic strategies. However, successful clinical translation will also require scalable manufacturing and clear regulatory pathways. Because electrospun drug-eluting meshes are typically classified as combination products, clinical adoption will depend on reproducible large-scale fabrication and coordinated evaluation of device biocompatibility, drug stability, and controlled release performance. Addressing these experimental, manufacturing, and regulatory challenges will be essential for transforming electrospun meshes from promising laboratory systems into clinically validated platforms for postoperative pain management.

Future progress in this field will depend on addressing two major challenges: expanding in vivo validation and establishing standardised evaluation frameworks. Most electrospun drug-delivery systems reported to date focus primarily on release kinetics or early pharmacological effects rather than long-term implant performance. Consequently, key translational questions including chronic tissue response, polymer degradation behaviour, mechanical stability, and sustained pharmacological efficacy over clinically relevant time frames—remain unexplored. In parallel, the development of standardised experimental protocols will be essential to improve cross-study comparability. Current investigations frequently emphasise release profiling, while pharmacokinetic measurements, behavioural pain assessment, and long-term histological analyses are reported inconsistently. Establishing unified evaluation endpoints would facilitate more rigorous comparison between electrospun meshes and conventional analgesic delivery strategies.

## 5. Conclusions

Electrospun membranes represent a highly promising platform for POPM by enabling localized, sustained, and multimodal drug delivery while minimizing systemic exposure and associated adverse effects. Their tuneable fibrous architecture, broad polymer compatibility, and capacity for incorporating analgesic, anti-inflammatory, and adjunctive bioactive agents provide clear advantages over conventional administration routes. Advances in polymer selection, blending strategies, and multilayer or core–shell electrospinning have enabled increasingly precise control over drug-release kinetics, allowing therapeutic profiles to be aligned with the temporal evolution of postoperative pain and tissue healing. Nevertheless, significant challenges remain before widespread clinical translation can be achieved. Control of initial burst release, prediction of long-term in vivo behaviour, and reproducible scaling of complex electrospun architectures under Good Manufacturing Practice conditions remain unresolved. In addition, mechanical performance is inconsistently reported and rarely benchmarked against application-specific clinical requirements, limiting cross-study comparability and rational device selection. From a regulatory perspective, electrospun drug-eluting membranes are typically classified as combination products, necessitating coordinated evaluation of both the device and pharmacological components. Future research should therefore prioritize standardised mechanical and biological evaluation frameworks, clinically relevant in vivo models, and scalable manufacturing strategies alongside continued materials and formulation innovation. Addressing these translational constraints will be essential for enabling electrospun membranes to progress from promising experimental systems to clinically validated tools for effective POPM.

## Figures and Tables

**Table 1:** Advantages and disadvantages of various drug administration routes.

Route	Advantages	Disadvantages	Ref.
Intravenous	Provides immediate and complete systemic bioavailability with rapid onset of action; allows precise titration of plasma drug concentrations; ideal for emergency and critical care applications.	Highly invasive and irreversible once administered; associated with risks of infection, thrombophlebitis, embolism, and vascular irritation; requires aseptic technique and skilled personnel.	[22,23]
Epidural	Enables regional anesthesia or analgesia with minimal systemic exposure; allows continuous infusion for prolonged pain management; suitable for postoperative or obstetric applications.	Technically demanding; potential complications include dural puncture, infection, or nerve injury; variable spread of drug within the epidural space.	[24,25]
Local	Provides targeted or systemic delivery of drugs with controlled absorption kinetics; suitable for compounds unstable in the gastrointestinal tract or with poor oral bioavailability.	Invasive and potentially painful; risk of infection, bleeding, or local tissue damage; requires sterile preparation and administration by trained personnel.	[26,27]
Oral	Non-invasive, convenient, and cost-effective; suitable for chronic therapy and self-administration; high patient compliance.	Subject to variable absorption and extensive first-pass metabolism; degradation by gastric acid or digestive enzymes; onset of action relatively slow.	[28]
Inhalational	Provides rapid onset through the extensive pulmonary surface area; enables high local concentrations for respiratory therapy or systemic absorption for selected drugs.	Efficacy dependent on inhalation technique and device performance; particle deposition may be inconsistent; risk of airway irritation or bronchospasm.	[29]
Transdermal	Non-invasive and patient-friendly; bypasses hepatic first-pass metabolism; allows sustained, controlled, or localized drug release over extended periods.	Limited to lipophilic, low-molecular-weight drugs due to barrier properties of the stratum corneum; potential for skin irritation or contact sensitization.	[30,31]
Rectal	Useful when oral administration is contraindicated; partially avoids first-pass hepatic metabolism; permits both local and systemic effects.	Absorption is variable and formulation-dependent; potential for discomfort, leakage, and poor patient acceptance.	[32]

**Table 2:** Aspects of selected drugs used in POPM.

Drug	Mechanism of Action	Half-Life	Administration	Advantages	Disadvantages	Ref.
Morphine	Acts on mu-opioid receptors in the CNS to inhibit pain transmission.	2–4 h	Oral, IV, Epidural, PCA	Potent analgesic, widely available, effective for severe pain	Risk of respiratory depression, constipation, addiction potential	[44]
Fentanyl	Binds to μ opioid receptors in the CNS, similar to morphine but more potent.	3–7 h	IV, Transdermal, Epidural, PCA	Potent, fast-acting, less histamine release (less pruritus)	Risk of respiratory depression, dependency, must be carefully dosed	[45]
Ibuprofen	Inhibits COX-1 and COX-2 enzymes, reducing prostaglandin production, decreasing pain and inflammation.	2–4 h	Oral, IV	Effective for mild to moderate pain, anti-inflammatory properties	Gastrointestinal side effects (bleeding, ulcers), renal toxicity	[46]
Naproxen	Inhibits COX enzymes, reducing prostaglandin production.	12–17 h	Oral	Long-lasting pain relief, effective anti-inflammatory	Risk of gastrointestinal issues, renal impairment, cardiovascular risks	[47]
Acetaminophen	Inhibits COX enzymes in the brain, modulating pain perception.	2–3 h	Oral, IV	Generally well-tolerated, low risk of gastrointestinal issues	Liver toxicity in overdose, less effective for severe pain	[38]
Lidocaine	Blocks sodium channels in nerves, preventing pain signal transmission.	1–2 h (IV), 2–3 h (Epidural)	IV, Epidural, Peripheral Nerve Block	Rapid onset, effective for localized pain, minimal systemic side effects	Short duration, risk of toxicity with high doses	[40]
Bupivacaine	Blocks sodium channels in nerves, providing local anaesthetic effect.	3–6 h	Epidural, Peripheral, Nerve Block	Long-lasting pain relief, effective for regional anaesthesia	Cardiotoxicity at high doses, slower onset compared to lidocaine	[48]
Gabapentin	Inhibits excitatory neurotransmitter release by binding to calcium channels.	5–7 h	Oral	Helpful for neuropathic pain, can enhance effects of other analgesics	Sedation, dizziness, peripheral edema	[49]
Amitriptyline	Inhibits reuptake of serotonin and norepinephrine, modulating pain pathways.	10–50 h	Oral	Effective for neuropathic pain, can improve mood and sleep	Sedation, anticholinergic effects (dry mouth, constipation)	[50]
Duloxetine	Inhibits reuptake of serotonin and norepinephrine, affecting pain processing pathways.	12 h	Oral	Effective for neuropathic pain and depression, well-tolerated	Nausea, fatigue, potential for serotonin syndrome	[51]

**Table 3:** Selected studies on electrospun drug carrier systems. N/A: Not available.

Drug	Polymer	Polymer Wettability	Mechanical Studies	Drug Release Studies	Animal Model	Observation Time Points	Ref
Lidocaine, Diclofenac	Carboxymethylcellulose (CMC)/polyethylene oxide (PEO)	Hydrophilic	N/A	UV-Vis spectrophotometry	N/A	N/A	[115]
Lidocaine, Diclofenac sodium	Carboxymethyl cellulose (CMC)/alginate (ALG)/polyethylene oxide (PEO)	Hydrophilic	N/A	UV-Vis spectrophotometry	N/A	N/A	[116]
Lidocaine, Epinephrine	Poly(ε-caprolactone) (PCL)/Poly[(d,l)-lactide-*co*-glycolide] (PLGA) (lactide/glycolide: 50/50)	Hydrophobic	UTM	HPLC	New Zealand white rabbits	Days 1, 2, 4, 7, 10 and 14	[101]
Lidocaine hydrochloride	Poly([d,l]-lactide-*co*-glycolide) (PLGA)	Hydrophobic	N/A	HPLC	Wistar rats	1, 3, 7, 10 and 14 days	[102]
Lidocaine, human EGF (hEGF)	Poly[(d,l)-lactide-co-glycolide] (PLGA)	Hydrophobic	UTM	HPLC	Wistar rats	Days 1, 3, 7, and 14; blood was collected from the rats, and lidocaine and hEGF concentrations were measured.	[103]
Lidocaine, Metronidazole, Oestradiol	Polycaprolactone (PCL)/poly(lactic-co-glycolic acid) (PLGA)	Hydrophobic	UTM	HPLC	Sprague-Dawley rats	Days 1, 4, 7, and 28	[104]
Lidocaine hydrochloride, Ketorolac	Poly[(d,l)-lactide-*co*-glycolide] (PLGA)	Hydrophobic	UTM	HPLC	Wistar rats	Day 7	[114]
Lidocaine, Mupirocin	Polycaprolactone (PCL)/Chitosan	Mixed	Dynamic mechanical testing	microvolume UV-Vis spectrophotometry	N/A	N/A	[77]
Lidocaine hydrochloride	Poly(vinylpyrrolidone) (PVP)/Eudragit RS100/poly(caprolactone) (PCL)/poly(ethylene oxide) (PEO)	Mixed	N/A	RP-HPLC	N/A	N/A	[78]
Lidocaine hydrochloride, Vancomycin hydrochloride, Ceftazidime hydrate	Poly(lactic-co-glycolic acid) (PLGA)	Hydrophobic	UTM	HPLC	New Zealand White Rabbits	2, 7, 14, 28 days	[79]
Bupivacaine hydrochloride	Poly (D, L-lactic-*co*-glycolic acid) (PLGA)	Hydrophobic	N/A	HPLC	Rats	Drug plasma concentrations were measured from day 1 to day 35	[80]
Bupivacaine hydrochloride, Acetaminophen	Poly(lactic-co-glycolic acid) (PLGA)/Polyethylene glycol (PEG)	Mixed	N/A	HPLC	Rats	Days 1, 3, 7, 14, 21, 28, and 35	[81]
Bupivacaine, Doxycycline	Poly(lactic-co-glycolic acid) (PLGA)/Collagen	Mixed	UTM	HPLC	Sprague Dawley Rats	1, 3, 7, 14, 21, 28 days	[82]
Bupivacaine, Indomethacin	Poly(lactic-co-glycolic acid) (PLGA)	Hydrophobic	UTM	HPLC	Male Sprague Dawley rats	4 weeks	[83]
Bupivacaine, Celecoxib	Poly(lactic-co-glycolic acid) (PLGA)	Hydrophobic	UTM	HPLC	Male Sprague Dawley rats	4 weeks	[84]
Ketorolac, Acyclovir	Poly(lactic-co-glycolic acid) (PLGA)	Hydrophobic	UTM	HPLC	Sprague-Dawley rats	1, 3, 7, and 14 days	[85]
Ketorolac, Ceftazidime, Vancomycin	Poly(lactic-co-glycolic acid) (PLGA)	Hydrophobic	UTM	HPLC	Wistar rats	Days 1, 3, 7, 14, 21, and 28	[86]
Meloxicam	Chitosan, Polyvinyl alcohol (PVA)/Hydroxyapatite (HA)	Hydrophilic	N/A	UV-Vis spectrophotometry	N/A	N/A	[87]
Meloxicam, Mitomycin-C	Polycaprolactone (PCL)/Chitosan (CS)	Mixed	N/A	UV-Vis spectrophotometry	New Zealand Rabbits	4 weeks	[88]
Indomethacin	Polyvinylpyrrolidone (PVP)	Hydrophilic	N/A	HPLC	N/A	N/A	[89]
Indomethacin	Gelatin and Poly(L-lactide-co-caprolactone) (PLCL)	Mixed	UTM	RP-HPLC	N/A	N/A	[90]
Aceclofenac	Polyvinylpyrrolidone (PVP, Luviskol^®^)/polyethylene glycol 300 (PEG 300)	Hydrophilic	Texture analyser	HPLC	New Zealand rabbits	0, 0.5, 1, 1.5, 2, 2.5, 3, 4, 5, 6, and 8 h	[91]
Ibuprofen	Polyvinyl pyrrolidone (PVP K30)/hydroxypropyl methyl cellulose (HPMC K4M)	Hydrophilic	N/A	UV-Vis spectrophotometry	Sprague-Dawley rats	Once a day for 4 days	[92]
Acetaminophen, Gabapentin	Polyethylene oxide (PEO)/poly(vinyl alcohol) (PVA)/sodium alginate (low-medium viscosity)	Hydrophilic	N/A	UV-Vis spectrophotometry (Dual beam)	N/A	N/A	[93]
Benzocaine	Cellulose acetate	Hydrophobic	N/A	Automated Transdermal Diffusion Cells Sampling System/ UV–VIS spectrophotometer	N/A	N/A	[94]
Benzocaine	Cellulose Acetate	Hydrophobic	N/A	UV-Vis spectrophotometry	N/A	N/A	[94]
Ropivacaine	Poly(caprolactone) (PCL)	Hydrophobic	N/A	LC-MS	Sprague Dawley (SD) rats	2, 4, 6, 12, 24, 48, 72 h, post-surgery	[95]
Buprenorphine hydrochloride	Poly(vinyl pyrrolidone) (PVP)/poly(vinyl alcohol) (PVA)	Hydrophilic	5566 Instron universal testing machine	HPLC	N/A	N/A	[96]
Naproxen	Ethyl Cellulose (EC)/Poly(vinyl) pyrrolidone (PVP)	Mixed	N/A	UV-Vis spectrophotometry	N/A	N/A	[97]
	Thermoplastic polyurethane (TPU) β-Cyclodextrin (β-CD)/2-Hydroxypropyl-β-cyclodextrin (HP-β-CD)	Mixed	N/A	N/A	N/A	N/A	[98]
	Poly(lactic-co-glycolic acid) (PLGA)	Hydrophobic	Tensile ramp to failure	UV-Vis spectrophotometry	Sprague Dawley Rats	0.5, 3, 7, 14 days	[99]
Curcumin	Polycaprolactone (PCL, MW 80,000)/gelatin (type A)	Mixed	N/A	HPLC	N/A	N/A	[100]
Capsaicin, Gentamicin	Poly(vinyl alcohol) (PVA)/gelatin (type A, from porcine skin)	Hydrophilic	N/A	MMR	Rats	Rats were sacrificed after 21 days	[105]
COX-2 (human) polyclonal antibody	Polyaniline/polystyrene	Hydrophobic	N/A	N/A	N/A	N/A	[106]
Tramadol hydrochloride	Poly(ε-caprolactone) (PCL)/Polyethylene oxide (PEO)	Mixed	N/A	UV-Vis spectrophotometry	N/A	N/A	[107]
Neostigmine methylsulfate	Poly vinyl alcohol (PVA)	Hydrophilic	N/A	UV-Vis spectrophotometry	Sprague–Dawley rats	1, 5 and 24 h after injection and then were sampled once weekly for 4 weeks	[108]
Gabapentin	Cellulose acetate (CA)/gelatin (Gel)	Mixed	Uniaxial tensile testing device	N/A	Wistar rats	48 h, 72 h and 96 h	[109]
Diclofenac Sodium	Chitosan iodoacetamide (CI)/Polyvinyl Alcohol (PVA)	Hydrophilic	N/A	MMR	Male rats	21 days	[105]
Borneol	Poly(L-lactic acid) (PLLA)/Cellulose acetate butyrate (CAB)	Hydrophobic	N/A	Emission Measurements	N/A	N/A	[110]
Gelsevirine	Polycaprolactone (PCL)/Gelatin/Sodium Alginate (SA)/Silk Fibroin (SF)	Mixed	Multifunction tensile testing machine (WDW-1)	HPLC	Male C57BL/6 mice	3, 7, 14, 21 days	[111]
Papaverine	Poly-l-lactic acid/Polyethylene glycol (PLLA/PEG)	Mixed	Purpose-built mechanical testing machine	UV-Vis spectrophotometry	New Zealand Rabbits	14 days	[112]
Lovastatin	Polycaprolactone (PCL)/Silk Fibroin (SF)	Hydrophobic	Electronic universal testing machine	UHPLC	Male BALB/c mice	7, 14, 30 days	[113]

**Table 4:** Selected studies and their drug-release rates at fixed intervals. N/A: Not available.

Polymer	Ingredient	Release Configuration	Drug Release				Ref.
			25%	50%	75%	100%	
Carboxymethylcellulose (CMC)/Polyethylene oxide (PEO)	Lidocaine, Diclofenac	DIC	50 min	250 min	350 min	>3000 min	[115]
Carboxymethyl cellulose (CMC)/alginate (ALG)/polyethylene oxide (PEO)	Lidocaine, Diclofenac	DIC	50 min	435 min	1700 min	>3000 min	[116]
Poly([d,l]-lactide-co-glycolide) (PLGA)	Lidocaine, Epinephrine	Epinephrine	5 days	7.5 days	17.5 days	>27 days	[101]
Poly([d,l]-lactide-co-glycolide) (PLGA)	Lidocaine hydrochloride	N/A	>24 h	3 days	7 days	15 days	[102]
Poly([d,l]-lactide-co-glycolide) (PLGA)	Lidocaine, human EGF (hEGF)	N/A	1–2 days	3–5 days	10 days	30 days	[103]
Polycaprolactone (PCL)/poly(lactic-*co*-glycolic acid) (PLGA)	Lidocaine, Metronidazole, Oestradiol	Metronidazole	0.3 days	0.4 days	0.8 days	2.5 days	[104]
		Lidocaine	/	/	/	20 days	[104]
Poly[(d,l)-lactide-*co*-glycolide] (PLGA)	Lidocaine hydrochloride, Ketorolac	Lidocaine	0.1 days	0.1 days	1 day	22.5 days	[114]
Polycaprolactone (PCL)/Chitosan	Lidocaine, Mupirocin	Lidocaine	0.4 h	0.5 h	2 h	>6 h	[77]
Poly(vinylpyrrolidone) (PVP)/Eudragit RS100 Poly(caprolactone) (PCL)/poly(ethylene oxide) (PEO)	Lidocaine hydrochloride	N/A	15 min	30 min	1 h	1–2 h	[78]
(PLGA) Poly (D, L-lactic-*co*-glycolic acid)	Bupivacaine hydrochloride	N/A	>24 h	5–7 days	10–15 days	35 days	[80]
(PLGA) Poly (lactic-*co*-glycolic acid) PEG	Bupivacaine hydrochloride Acetaminophen	Gel/fibre: ACM	2 days	7 days	15 days	20 days	[81]
Poly(lactic-co-glycolic acid) (PLGA), Collagen	Bupivacaine, Doxycycline	Doxycycline	0.7 days	13.5 days	/	/	[82]
		Bupivacaine	0.4 days	0.7 days	10 days	/	[82]
Poly(lactic-co-glycolic acid) (PLGA)	Bupivacaine, Indomethacin	Indomethacin	0.3 days	0.7 days	1.1 days	2.5 days	[83]
		Bupivacaine	0.7 days	1.1 days	8.5 days	/	[83]
Poly(lactic-co-glycolic acid) (PLGA)	Bupivacaine, Celecoxib	Celecoxib	0.8 days	3 days	/	/	[84]
		Bupivacaine	>24 h	3 days	7 days	28 days	[84]
		Ketorolac	0.1 days	1 day	3 days	25 days	[84]
Poly(lactic-co-glycolic acid) (PLGA)	Ketorolac, Acyclovir	Acyclovir	0.8 days	1 day	6.5 days	21 days	[85]
		Ketorolac	0.8 days	1 day	3 days	21 days	[85]
Poly(lactic-co-glycolic acid) (PLGA)	Ketorolac, Ceftazidime, Vancomycin	Ketorolac	0.5 days	0.7 days	1 day	>30 days	[86]
Chitosan, Polyvinyl alcohol (PVA), Hydroxyapatite (HA)	Meloxicam		0.5 h	1 h	4 h	25 h	[87]
Polycaprolactone (PCL) and Chitosan (CS)	Meloxicam, Mitomycin-C	Meloxicam	2 days	4 days	7 days	>14 days	[88]
		Meloxicam/Mitomycin-C	2 days	4.5 days	8.5 days	>14 days	[88]
Polyvinyl pyrrolidone (PVP)	Ibuprofen	Sustained-release fibres	0	50 min	250 min	>1000 min	[92]
Poly(lactic-co-glycolic acid) (PLGA)	Ibuprofen	N/A	2.5 days	24 days	50 days	>60 days	[99]
Polyethylene oxide (PEO)	Gabapentin	First layer	6 min	17 min	35 min	>90 min	[93]
PEO/Sodium Alginate	Acetaminophen	Second layer	10 min	35 min	70 min	>180 min	[93]
Cellulose acetate	Benzocaine	pH-9	30 min	180 min	300 min	>1500 min	[94]
Poly(ε-caprolactone) (PCL)/polyethylene oxide (PEO)	Tramadol hydrochloride	N/A	>24 h	3 days	6 days	10 days	[107]
Poly(L-Lactic Acid) (PLLA), Cellulose Acetate Butyrate (CAB)	Borneol	PLLA	0.4 h	0.7 h	1.5 h	/	[110]
Polycaprolactone (PCL) and Silk Fibroin (SF)	Lovastatin	RLPS (random lovastatin-loaded PCL/SF nanofibres)	70 h	/	/	/	[113]
		ALPS (aligned lovastatin-loaded PCL/SF nanofibres)	120 h	/	/	/	[113]
Gelatin and Poly(L-lactide-co-caprolactone) (PLCL)	Indomethacin		1 day	3 days	6 days	14 days	[90]
Chitosan iodoacetamide (CI), Polyvinyl Alcohol (PVA)	Diclofenac sodium		0.5 h	4.5 h	48 h	>48 h	[105]
Poly(vinyl pyrrolidone) (PVP)/poly(vinyl alcohol) (PVA)	Buprenorphine hydrochloride	Cross-linked Bup/PVP/PVA	3 h	24 h	/	/	[96]
Ethyl Cellulose (EC)/Poly(vinyl) pyrrolidone (PVP)	Naproxen	N/A	10 h	22.5 h	45 h	>80 h	[97]
Thermoplastic polyurethane (TPU) β-Cyclodextrin (β-CD)/2-Hydroxypropyl-β-cyclodextrin (HP-β-CD)	Naproxen	N/A	/	30–45 min	1.3 h	4 h	[98]
Carboxymethyl cellulose (CMC)/alginate (ALG)/polyethylene oxide (PEO)	Ibuprofen Diclofenac sodium	LID	200 min	300 min	1700 min	>3000 min	[116]
		DICS	0	0	125 min	>3000 min	[116]
Poly([d,l]-lactide-co-glycolide) (PLGA)		E (2:1)	4.75 days	8 days	17 days	27.5 days	[101]
		E (3:1)	2.5 days	10 days	17 days	>30 days	[101]
		E (6:1)	1 day	2 days	5 days	25 days	[101]
		LID (2:1)	1 day	13.5 days	24 days	>30 days	[101]
		LID (3:1)	6 days	11 days	17.5 days	27.5 days	[101]
		LID (6:1)	3 days	11.5 days	17.5 days	22.5 days	[101]
Polyvinyl pyrrolidone (PVP K30)/hydroxypropyl methyl cellulose (HPMC K4M)	Ibuprofen	Fast-release fibres	2 min	3.5 min	5 min	>30 min	[92]
Polycaprolactone (PCL)	Curcumin	N/A	0.4 day	1 day	7 days	>36 days	[100]
Polyethylene oxide (PEO)/poly(vinyl alcohol) (PVA)/sodium alginate (low-medium viscosity)	Acetaminophen, Gabapentin	Second layer PVA-SA-NCR	2 h	4 h	6 h	>18 h	[93]
		Second layer PVA-SA-ICL	5 h	8.5 h	/	/	[93]
Poly(vinyl pyrrolidone) (PVP)/poly(vinyl alcohol) (PVA)	Buprenorphine hydrochloride	Bup/PVP	0.37 h	24 h	/	/	[96]
		Bup/PVP/PVA	1.25 h	96 h	/	/	[96]
Polycaprolactone (PCL)/poly(lactic-co-glycolic acid) (PLGA)		Estradiol	28 days	>30 days	>30 days	>30 days	[104]
		Lidocaine	/	/	/	/	[104]
		Metronidazole	/	/	/	2.5 days	[104]
Poly(lactic-co-glycolic acid) (PLGA)/Polyethylene glycol (PEG)	Bupivacaine hydrochloride, Acetaminophen	Fibre: ACM	>24 h	5 days	10 days	14 days	[81]
		Gel/fibre: Bup	>12 h	1 day	7 days	14 days	[81]
		Fibre: Bup	>24 h	3 days	10 days	14 days	[81]
Poly(vinyl alcohol) (PVA)/gelatin (type A, from porcine skin)	Capsaicin, Gentamicin	N/A	>30 min	1 h	1.5 h	2 h	[105]
Poly(lactic-co-glycolic acid) (PLGA)	Ketorolac, Ceftazidime, Vancomycin	Ketorolac	/	0.5 days	2.2 days	/	[86]
		Ceftazidime	0.7 days	6 days	11 days	>30 days	[86]
		Vancomycin	1 day	9 days	/	/	[86]
Cellulose acetate	Benzocaine	pH-7.4	190 min	320 min	/	/	[94]
		pH-3.7	250 min	/	/	/	[94]
Polycaprolactone (PCL)/Chitosan	Mupirocin, Lidocaine	Mupirocin	2 h	8 h	24 h	>120 h	[77]
		Lidocaine	0.4 h	0.5 h	3 h	/	[77]
Polycaprolactone (PCL)/Chitosan (CS)	Mitomycin-C	Mitomycin-C	2 days	4 days	9 days	>14 days	[88]
Poly(L-lactic acid) (PLLA)/cellulose acetate butyrate (CAB)	Borneol	PLLA/CAB (30%)	0.8 h	1.7 h	3 h	/	[110]
		PLLA/CAB (70%)	2.2 h	8 h	/	/	[110]
Poly(lactic-co-glycolic acid) (PLGA)	Celecoxib	PLGA: Celecoxib (6:1)	0.3 days	5 days	/	/	[84]
		PLGA: Celecoxib (4:1)	0.2 days	2.5 days	/	/	[84]
Polycaprolactone (PCL)/gelatin/sodium alginate (SA)/silk fibroin (SF)	Gelsevirine		2 h	9 h	55 h	/	[111]
Poly-L-lactic acid (PLLA)/Polyethylene glycol (PEG)	Papaverine		0.5 day	2 days	14 days	/	[112]

## Data Availability

No new data were created or analysed in this study. Data sharing is not applicable to this article.

## Supplementary Materials

The following supporting information can be downloaded at: https://www.mdpi.com/article/10.3390/pharmaceutics18050538/s1, Supplementary Material S1: Search Strategy and Study Selection.

## Abbreviations

Abbreviation	Full term
ALG	Alginate
CAB	Cellulose acetate butyrate
CA	Cellulose acetate
CI	Chitosan iodoacetamide
CMC	Carboxymethyl cellulose
CS	Chitosan
ERAS	Enhanced Recovery After Surgery
FTIR	Fourier-transform infrared spectroscopy
GBP	Gabapentin
Gel	Gelatin
HA	Hydroxyapatite
hEGF	Human epidermal growth factor
HPLC	High-performance liquid chromatography
NSAIDs	Non-steroidal anti-inflammatory drugs
PBS	Phosphate-buffered saline
PBAT	Poly(butylene adipate-co-terephthalate)
PCL	Polycaprolactone
PDLLA	Poly(D,L-lactic acid)
PEG	Poly(ethylene glycol)
PEG 300	Polyethylene glycol 300
PEO	Poly(ethylene oxide)
P3B	Poly(3-hydroxybutyrate)
LA	Poly(lactic acid)
PLCL	Poly(lactide-co-caprolactone)
PLGA	Poly(lactic-co-glycolic acid)
PLLA	Poly(L-lactic acid)
POPM	Postoperative pain management
PRISMA	Preferred Reporting Items for Systematic Reviews and Meta-Analyses
PVA	Poly(vinyl alcohol)
PVP	Poly(vinylpyrrolidone)
RP-HPLC	Reverse-phase high-performance liquid chromatography
SA	Sodium alginate
SEM	Scanning electron microscopy
SF	Silk fibroin
TPU	Thermoplastic polyurethane
UV	Ultraviolet
UV–Vis	Ultraviolet–visible spectroscopy
UHPLC	Ultra-High-Performance Liquid Chromatography
DAD	Diode array detection
MMR	Multimode microplate reader
LC-MS	Liquid Chromatography–Mass Spectrometry
UTM	Universal Testing Machine
