# Supplementary material for: The Potential of Electrospun Meshes in Postoperative Pain Management
Source: Pharmaceutics. 2026 Apr 28;18(5):538. doi: 10.3390/pharmaceutics18050538 (PMC13210733; doi:10.3390/pharmaceutics18050538)
Supplement: Supplementary file 1 [file pharmaceutics-18-00538-s001.zip › pharmaceutics-4103923-supplementary.pdf]

Supplementary Material S1. Search Strategy and Study Selection

Electrospun Meshes for Postoperative Pain Management (Systematic Review)

1. Reporting Standard

This systematic review was conducted and reported in accordance with the PRISMA 2020 Statement. The study identification, screening, eligibility assessment, and inclusion processes are summarized in the PRISMA 2020 flow diagram (Figure 1). Due to the predominantly preclinical, heterogeneous, and non-comparative nature of the included studies, formal risk-of-bias assessment and quantitative meta-analysis were not applicable

2. Information Sources

Electronic literature searches were conducted from database inception to the final search date ([insert final search date]) using the following databases:

- PubMed/MEDLINE
- Scopus
- Web of Science Collection (Science Citation Index Expanded (SCIE), Social Sciences Citation Index (SSCI), Arts & Humanities Citation Index (AHCI), Emerging Sources Citation Index (ESCI), Book Citation Index (BCI), Conference Proceedings Citation Index (CPCI), Chinese Science, Citation Database, SciELO Citation Index, Korea Citation Index, Russian Science Citation Index, Arabic Regional Citation Index)

No trial registries were searched. Grey literature sources and manual reference list screening were not used as additional identification methods.

3. Search Strategy Development

The search strategy was developed a priori using a combination of free-text terms and controlled vocabulary where applicable. The strategy targeted three conceptual domains: (i) electrospinning and nanofibrous systems; (ii) mesh or scaffold constructs; and (iii) postoperative pain management and analgesia. Boolean operators, truncation, and phrase searching were applied as appropriate to each database.

4. Full Electronic Search Strategy

The complete electronic search strategies, as executed, are reported below. Database-specific syntax modifications were applied while maintaining conceptual equivalence.

Database	Search string (executed)
PubMed/MEDLINE	(electrospun OR electrospinning OR nanofiber* OR nanofibrous) AND (mesh* OR scaffold* OR mat*) AND (postoperative OR post-operative OR surgical) AND (pain OR analgesia OR analgesic OR "local

	anesthetic*" OR lidocaine OR bupivacaine OR ropivacaine)
Scopus	TITLE-ABS-KEY((electrospun OR electrospinning OR nanofiber* OR nanofibrous) AND (mesh* OR scaffold* OR mat*) AND (postoperative OR post-operative OR surgical) AND (pain OR analgesia OR analgesic OR "local anesthetic*" OR lidocaine OR bupivacaine OR ropivacaine))
Web of Science Core Collection	TS=((electrospun OR electrospinning OR nanofiber* OR nanofibrous) AND (mesh* OR scaffold* OR mat*) AND (postoperative OR post-operative OR surgical) AND (pain OR analgesia OR analgesic OR "local anesthetic*" OR lidocaine OR bupivacaine OR ropivacaine))

No study design filters were applied during database searching to avoid excluding potentially relevant preclinical, translational, or clinical studies.

5. Eligibility Criteria

Inclusion criteria:

- Electrospun nanofibrous meshes or scaffolds intended for perioperative or postoperative application.
- Evaluation of pain-related outcomes and/or local analgesic delivery.
- Original experimental studies (in vitro, in vivo, translational, or clinical).
- Full-text articles published in peer-reviewed journals.

Exclusion criteria:

- Non-electrospinning-based systems or absence of nanofibrous architecture.
- Irrelevant title or abstract with no clear postoperative pain management relevance.
- Lack of objective or comprehensive outcome data after full-text assessment.
- Conference abstracts or reports without accessible full text.

6. Study Selection and Screening

Records were screened in a two-stage process. Titles were initially screened for relevance, followed by abstract assessment. Full-text manuscripts were subsequently evaluated for eligibility. Reasons for exclusion at each stage were documented.

7. PRISMA Flow Summary

The PRISMA 2020 flow diagram reports the following study selection results:

- Records identified through databases: n = 10; registers: n = 0.
- Records removed before screening for other reasons: n = 2; duplicates removed: n = 0.
- Records screened by title: n = 172; records excluded due to irrelevant title: n = 120.

- Reports sought for retrieval: n = 52; reports not retrieved after abstract assessment: n = 4.
- Reports assessed for eligibility: n = 48; reports excluded after full-text evaluation due to lack of objective or comprehensive data: n = 4.
- Studies included in the final review: n = 44.

8. Reproducibility

The full electronic search strategies are reported to enable reproducibility. Any future updates to the searches or amendments to eligibility criteria should be documented accordingly.
